# Tissue dissolution and modifications in dentin composition by different sodium hypochlorite concentrations

**DOI:** 10.1590/1678-775720150524

**Published:** 2016

**Authors:** Talita TARTARI, Luciano BACHMANN, Amanda Garcia Alves MALIZA, Flaviana Bombarda ANDRADE, Marco Antonio Hungaro DUARTE, Clovis Monteiro BRAMANTE

**Affiliations:** 1 - Universidade de São Paulo, Faculdade de Odontologia de Bauru, Departamento de Dentística, Endodontia e Materiais Odontológicos, Bauru, SP, Brasil.; 2 - Universidade de São Paulo, Faculdade de Filosofia, Ciências e Letras de Ribeirão Preto, Departamento de Física, Ribeirão Preto, SP, Brasil.

**Keywords:** Dentin, Dissolution, Fourier transform infrared spectroscopy, Organic matter, Sodium hypochlorite

## Abstract

Sodium hypochlorite (NaOCl) remains the most used irrigation solution during root canal preparation because of characteristics such as wide-spectrum antimicrobial activity and organic tissue dissolution capacity. However, these solutions can alter dentin composition and there is no consensus on the optimal concentration of NaOCl to be used.

**Objectives:**

To determine the organic matter dissolution and changes in dentin chemical composition promoted by different concentrations of NaOCl over time. Material and Methods: Fragments of bovine muscle tissue were weighed before and after 5, 10, and 15 min of immersion in the groups (n=10): G1- 0.9% saline solution; G2- 1% NaOCl; G3- 2.5% NaOCl; and G4- 5% NaOCl. Bovine dentin fragments were subjected to the same irrigants and absorption spectra were collected by Attenuated Total Reflectance of Fourier Transform Infrared Spectroscopy (ATR-FTIR) before and after 0,5, 1, 2, 3, 5, 8, and 10 min of immersion in the solutions. The ratios of the amide III/phosphate and carbonate/phosphate absorption bands were determined. The tissue dissolution and carbonate/phosphate ratios were submitted to the two-way analysis of variance (ANOVA) with Tukey’s multiple-comparison test (α<0.05) and to the one-way analysis of variance with Tukey’s (α<0.05). The amide III/phosphate ratio was analyzed by Friedman test (α<0.05) and the Kruskal-Wallis test with Dunn’s post-hoc (α<0.05).

**Results:**

The increase in NaOCl concentration and contact time intensified the dissolution of organic matter and dentin collagen with reduction in the amide III/phosphate ratio. Significant differences between all groups (p<0.05) were observed in the dissolution of organic matter at 10 min and in the amide III/phosphate ratio between the saline solution and 5% NaOCl at 5 min. The carbonate/phosphate ratio decreased significantly in G2, G3, and G4 after 0,5 min of immersion (p<0.05), but more alterations did not occur in the subsequent periods (p>0.05). Intergroup differences were not observed in this ratio (p>0.05).

**Conclusions:**

The increase in the exposure time and in the concentration of NaOCl solution lead to an increase in the tissue dissolution and dentin collagen deproteination. Furthermore, some carbonate ions are removed from the dentin inorganic phase by the NaOCl.

## INTRODUCTION

The physical and chemical effects of the irrigation solutions used in endodontics are crucial for cleaning and disinfection, since studies have shown that a large number of root dentin walls remain untouched after biomechanical preparation^25^. Different auxiliary chemical agents have been proposed, however, sodium hypochlorite (NaOCl) solutions are the most widely used for endodontic procedures because of their characteristics such as wide-spectrum antimicrobial activity and organic tissue dissolution capacity^29^ ^,^ ^33^. However, there is no consensus regarding the ideal concentration of NaOCl to be used.

An increase in the number of microorganisms was observed when intracanal medicament was not used between the treatment sessions and this fact was assigned to the organic tissue that remained in the root canal and provided ideal conditions for bacterial growth^9^. Possible ways to improve the tissue dissolution by NaOCl are the increase in the pH^7^, the concentration and temperature of the solutions, ultrasonic agitation, and prolonged working time^29^ ^,^ ^32^. However, the increase in concentration of NaOCl solutions can lead to undesirable effects such as an increase in toxicity to the periapical tissues^13^.

NaOCl solutions can also act in the dentin changing its chemical composition^19^. In mineralized dentin, the collagen fibrils are encapsulated by apatite crystals, thus the dimensions of molecules that can penetrate in the dentin structure should be smaller^35^. NaOCl molecules can penetrate in the apatite-encapsulated collagen matrix because of their low molecular weight (74.4 Da)^35^, and as a nonspecific oxidizing and proteolytic agent, can oxidize the organic matrix, denature the collagen, and adversely affects the mechanical properties of dentin^24^ ^,^ ^34^. The effects of NaOCl solutions on the collagen of the dentin organic matrix may also affect the sealing ability and the adhesion of resin-based cements and root canal sealers that chemically bond to the dentinal collagen^17^ ^,^ ^22^.

In addition, with the technological advancement in endodontics, the biomechanical preparation phase is becoming faster, and the use of more concentrated irrigants for adequate sanitization is probably necessary. Therefore, it is important to know how much the increase in NaOCl concentration, with the objective to enhance sanitization, improves the organic matter dissolution without causing much undesirable alterations of the chemical composition of the dentin. The aim of the present study was to determine the dissolution capacity of organic matter and the chemical alterations on the composition of the dentin surface produced by different concentrations of NaOCl at different exposure times. The null hypothesis tested was that the different concentrations of NaOCl solutions have similar capacity of tissue dissolution and effects on dentin composition and act similarly over time.

## MATERIAL AND METHODS

### Irrigation solutions

Concentrated (10-15%) NaOCl solution (Sigma-Aldrich; St. Louis, MO, USA) was diluted in distilled water to produce solutions with 1%, 2.5%, and 5% concentrations that were confirmed by iodometric titration. The solutions obtained were stored, protected from the light in airtight plastic bottles in a refrigerator at 4°C, and removed one hour before the experiments to reach room temperature. A 0.9% physiological saline solution was used as a control. The pHs of the solutions were determined before the experiments using a calibrated pH meter.

### Tissue dissolution

Bovine muscle tissue was acquired on the day of the experiment and kept refrigerated in 100% humidity. The muscle was cut with scalpel blades in pieces with 2x2x6 mm (width x thickness x length) and the specimens obtained were weighed on the FX-300 electronic balance (A&D Company; Tokyo, Japan). To do the sample calibration, the data obtained were submitted to statistical analysis to verify and ensure that all groups were statistically similar before the beginning of the experiment. Next, the samples were submitted to one of the following solutions (n=10): G1– 0.9% physiological saline solution (control); G2– 1% NaOCl; G3– 2.5% NaOCl; and G4– 5% NaOCl.

Specimens from each group were immersed for 5 min in individual containers filled with 10 mL of the test solution. All the containers were placed in an ultrasonic tub to agitate the irrigants for 15 s per each minute. Next, the specimens were submerged in distilled water for 0,5 min to remove the irrigation solutions. They were then blotted with filter paper and re-weighed. This procedure was repeated 3 times to obtain data of 5, 10, and 15 min of immersion. The solutions were renewed before each immersion period to simulate clinical conditions and to prevent saturation. All test procedures were done at room temperature (25°C).

### ATR-FTIR

**Figure 1:**
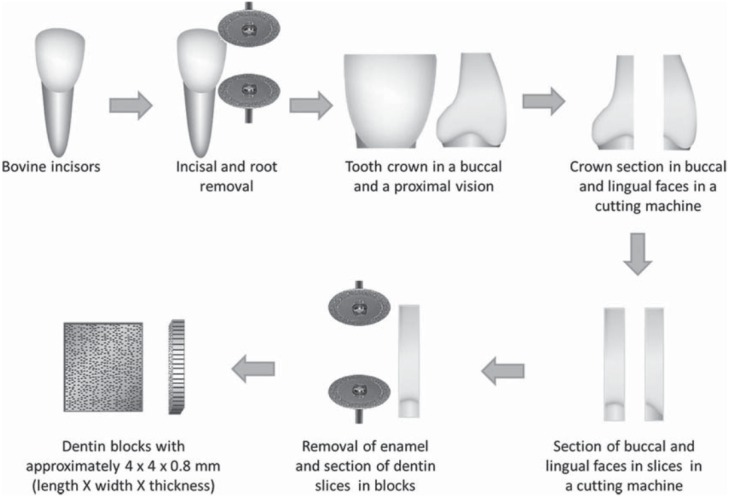
Sample preparation for the ATR-FTIR analysis

Crowns of bovine teeth were removed at the cementoenamel junction using a diamond disc at low-speed under water cooling. Then, the incisal of the crowns were removed in the same way. Each crown was then longitudinally sectioned in the mesiodistal direction in the Isomet 1000 cutting machine (Buehler Ltd.; Lake Bluff, IL, USA) to obtain the buccal and lingual portions. Slices with approximately 0.8 mm thicknesses were obtained from these crown halves. The slices were then cut again with a diamond disc at low-speed to remove the surrounding enamel and to obtain twenty specimens with approximately 4 mm x 4 mm x 0.8 mm (length x width x thickness) (Figure 1).

**Figure 2:**
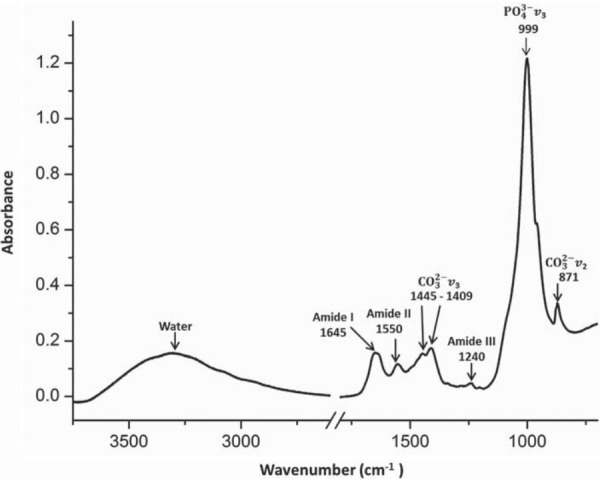
Absorbance spectrum of untreated dentin with the absorption peaks of the main dentin components

One surface of the dentin specimens was wet polished with 4000 grain silicon carbide abrasive papers (Buehler; Lake Bluff, IL, USA) and alpha alumina suspensions with 1 and 0.3 microns (Struers; Ballerup, Denmark) until a flat and smooth surface was obtained. Finally, the specimens were immersed in distilled water and ultrasonicated for 1 min to remove any residue from the polishing. They were then dried with absorbent paper and the polished surface positioned on the diamond crystal that was the internal reflection element from the Fourier Transform Infrared (FTIR) Spectrometer Nicolet 380 (Thermo Fisher Scientific Inc.; Waltham, MA, USA) and the absorbance spectra were collected by the technique of Attenuated Total Reflection (ATR), between wavenumbers of 4000 and 400 cm^-1^ at resolution of 1 cm^-1^ using 32 scans.

The specimens were randomly assigned to the previously described four groups (n=5). Each specimen was placed inside a microtube containing 1.5 mL of the solutions for 0,5 min and ultrasonicated for 15 s. Next, they were transferred to a microtube containing 1.5 mL of distilled water and rinsed for 1 min with 15 s of ultrasonic agitation. They were then dried with absorbent paper and the ATR-FTIR spectra recorded again. Specimens were replaced in the solutions for additional 0,5 min following the same protocol described to collect the new spectra. This process was sequentially repeated to obtain the spectra at time intervals of 0, 0.5, 1, 2, 3, 5, 8 and 10 min. However, after obtaining the 1 min spectrum, the ultrasonic agitation was performed for 15 s per each minute of immersion in the irrigants. To ensure the effectiveness of the solutions, they were renewed after the time intervals of 2, 5, and 8 min.

A typical absorbance spectrum obtained from a disc of untreated dentin is shown in Figure 2. In this spectrum the peaks between 3.750–750 cm^-1^ were identified. The areas of the absorption bands of phosphate (PO^3-^), carbonate (CO^3-^) , and amide III of each spectrum were determined. The wavenumber values employed for the area integrations for the amide III were between 1298–1216 cm^-1^ spectral range, between 888–816 cm^-1^ for the carbonate, and between 1170–780 cm^-1^ for the phosphate. Inside the phosphate spectral range there is the carbonate band at 888–816 cm^-1^, whose value was subtracted to obtain the real value of the area of phosphate band.

To evaluate the effects of NaOCl solutions on the chemical composition of dentin, two parameters were calculated. The first was the amide III/phosphate ratio that was used to determine the collagen deproteination by NaOCl. The amide III band was chosen, for in this region of 1298–1216 cm^-1^ there is no overlapping with bands of other dentin components. Instead, in bands of amides A and B at 3115 and 2860 cm^-1^ and amide I at 1645 cm^-1^, overlapping occurs with water bands, and in bands assigned to amide II, present at around 1550 cm^-1^, the overlapping occurs with carbonate bands^3^. The effect of NaOCl in the inorganic phase of the dentin was evaluated using the carbonate/phosphate (PO^3-^)/(CO^3-^)ratio, which was the second parameter. The ratios were obtained by taking the quotient between the areas of the bands.

The amide III/phosphate ratio was measured to evaluate how the amide III or the phosphate changed when immersed in NaOCl solution. For example, when this ratio decreases, it means that the amount of amide III (organic matter) decreased compared with the phosphate (inorganic matrix). However, when employing a chemical agent that removes organic matter and inorganic matter simultaneously, this ratio could stay unaltered. The carbonate/phosphate ratio is employed to evaluate the dissolution of the inorganic matrix; this ratio measures the carbonate dissolution in relation to the phosphate radical.

### Statistical analysis

The collected data of tissue dissolution and carbonate/phosphate ratios showed normal distribution, and were submitted to the two-way analysis of variance (ANOVA) with Tukey’s multiple-comparison test (α<0.05) to detect intragroup differences over time and the one-way analysis of variance with Tukey’s (α<0.05) to detect any differences between the groups at the same time period.

The amide III/phosphate ratio exhibited abnormal distribution. The nonparametric Friedman test (α<0.05) was used to detect intragroup differences among different periods of immersion and the Kruskal-Wallis test with Dunn’s *post-hoc* (α<0.05) test was used to detect intergroup differences in the same period.

## RESULTS

### Tissue dissolution

Table 1 presents the pHs of the solutions, mean value and standard deviation of the weight of fragments of bovine muscle tissue and percentage difference between the initial weight of the fragments and the weight after immersion in different solutions over time. The saline solution did not alter the weight of fragments between the periods analyzed (p>0.05). Tissue dissolution was directly dependent on the concentration of NaOCl solutions as well as the immersion time. The intragroup comparisons showed significant decrease in weight of the fragments for all immersion time periods in 1, 2.5, and 5% NaOCl (p<0.01). The intergroup comparison showed that the reduction in weights was higher with the increase in the concentration of NaOCl. Statistical differences between the groups were significant (p<0.01) in 5 min between G4 and all other groups, the G3 was equal to G2 but different from G1, and G2 was equal to G1. In 10 and 15 min of immersion, the intergroup differences were identified in the following order for tissue dissolution: G4>G3>G2>G1.

**Table 1:** pH of the different irrigation solutions and the mean (X) and standard deviation (SD) in mg of the weights of bovine muscle tissue fragments before and after different periods of immersion in the irrigators and the reduction in weight of the fragments in percentage.

GROUPS	pH	Initial weight	Weight after 5 min of immersion		Weight after 10 min of immersion		Weight after 15 min of immersion	
		X ± SD	X ± SD	Reduction in weight of the fragments (%)	X ± SD	Reduction in weight of the fragments (%)	X ± SD	Reduction in weight of the fragments (%)
G1- Saline	6.4	55.8 ± 1.8^A,a^	53.5 ± 3.1^A,a^	-4.12	54.5 ± 2.6^A,a^	-2.3	54.0 ± 2.7^A,a^	-3.2
G2- 1% NaOCl	11.7	55.9 ± 1.5^A,a^	50.9 ± 1.8A^B,b^	-8.9	44.1 ± 1.6^B,c^	-21.1	36.2 ± 2.1^B,d^	-35.2
G3- 2.5% NaOCl	12.05	54.7 ± 2.0^A,a^	48.8 ± 3.0^B,b^	-10.7	38.9 ± 2.1^C,c^	-28.8	29.6 ± 3.5^C,d^	-45.8
G4- 5% NaOCl	12.3	55.9 ± 2.2^A,a^	36.9 ± 3.0^C,b^	-33.9	21.8 ± 2.5^D,c^	-61	12.2 ± 1.5^D,d^	-78.1

Different lowercase letters in rows indicate statistically significant intragroup differences (Two-way Anova P<0.01); Different capital letters in columns indicate statistically significant intergroup differences in the same time period (One-way Anova p<0.01)

### ATR-FTIR

Table 2 presents the results of the amide III/phosphate ratio for dentin treated with irrigants. The saline solution did not alter this ratio between the periods analyzed (p>0.05). In G2, G3, and G4, the collagen was deproteinated by NaOCl solutions from the first period of immersion, resulting in decreases in the amide III/phosphate ratio. Intragroup significant differences (p<0.05) for the initial dentin composition were identified after 5 min of immersion in all NaOCl concentrations. There were no intergroup significant differences between all NaOCl concentrations in all periods analyzed (p>0.05), however, statistical differences were identified between the 5% NaOCl and the saline solution after 5 min of immersion. The effects of 1% and 2.5% NaOCl in the amide III/phosphate ratio were lower than the effects of 5% NaOCl, with no statistical differences (p>0.05) for the saline solution.

**Table 2:** Median (Med), minimum and maximum (Min – Max) values for the ratio of amide III/phosphate in dentin surface before and after immersion in the irrigation solutions in different periods of time. The ratio values are multiplied by 10-3.

GROUPS	Initial ratio	0.5 min	1 min	2 min	3 min	5 min	8 min	10 min
	Med	Med	Med	Med	Med	Med	Med	Med
	(Min - Max)	(Min - Max)	(Min - Max)	(Min - Max)	(Min - Max)	(Min - Max)	(Min - Max)	(Min - Max)
G1 - Saline solution	7.8 (5.3-9.9)^A,a^	8.2 (5.1-10.7)^A,a^	7.7 (5.5-11.1)^A,a^	7.8 (5.6-11.4)^A,a^	8.7 (5.0-12.0)^A,a^	8.6 (5.3-11.6)^A,a^	7.9 (6.0-11.7)^A,a^	8 (5.7-12.1)^A,a^
G2 - 1% NaOCl	6.3 (4.9-7.0)^A,a^	6.2 (4.6-7.0)^A,ab^	5.1 (3.7-6.9)^A,ab^	5.1 (3.5-6.5)^A,abc^	4.6 (3.7-6.0)^A,abc^	4.3 (3.5-6.1)^AB,bc^	4.1 (2.6-5.3)^AB,bc^	3.7 (2.0-5.0) ^AB,c^
G3 - 2.5% NaOCl	4.9 (4.0-9.1) ^A,a^	4.6 (3.7-8.8)^Aa^	3.9 (3.7-7.5) ^A,ab^	3.5 (3.4-7.8)^A,abc^	3.7 (3.2-6.9)^A,abc^	3.5 (2.9-6.3)^AB,bc^	3 (2.4-6.7)^AB,bc^	2.4 (2.0-5.9)^AB,c^
G4 - 5% NaOCl	6.7 (4.4-7.7)^A,a^	5.9 (3.6-7.0)^Aa^	5.1 (3.4-6.4) ^A,ab^	4.8 (3.1-5.9)^A,abc^	4.4 (3.0-5.6)^A,abc^	3.9 (2.2-4.5)^B,bc^	2.9 (1.5-3.3)^B,bc^	1.9 (1.2-2.9)^B,c^

Different lowercase letters in rows indicate statistically significant intragroup differences (Friedman p<0.05); Different capital letters in columns indicate statistically significant intergroup differences in the same time period (Kruskal-Wallis and Dunn *post-hoc* p<0.05)

Regarding the carbonate/phosphate ratio, all irrigants caused a decrease in its initial proportion (Table 3). However, only the NaOCl solutions produced significant intragroup changes (p<0.05) that were identified immediately after 0,5 min of immersion. Significant changes in this ratio were not observed between this time interval and the subsequent periods (p>0.05). Although the NaOCl solutions caused higher changes in the carbonate/phosphate ratio than saline solution, in the intergroup comparisons, no significant differences were identified between all groups in the periods analyzed (p>0.05).

**Table 3:** Mean (X) and standard deviation (SD) values for the ratio of carbonate/phosphate in dentin surface before and after immersion in the irrigation solutions in different periods of time.

GROUPS	Initial ratio	0.5 min	1 min	2 min	3 min	5 min	8 min	10 min
	(X ± SD)	(X ± SD)	(X ± SD)	(X ± SD)	(X ± SD)	(X ± SD)	(X ± SD)	(X ± SD)
G1 - Saline solution	19.3 ± 1.5^A,a^	19.3 ± 1.4^A,a^	19.3 ± 1.6^A,a^	19.0 ± 1.7^A,a^	18.8 ± 1.2^A,a^	19.0 ± 1.3^A,a^	18.9 ± 1.4^A,a^	19.1 ± 1.2^A,a^
G2 - 1% NaOCl	18.9 ± 1.7^A,a^	18.2 ± 1.5^A,b^	17.7 ± 1.7^A,b^	17.7 ± 1.6^A,b^	17.7 ± 2.0^A,b^	17.8 ± 1.7^A,b^	17.8 ± 1.6^A,b^	17.7 ± 1.6^A,b^
G3 - 2.5% NaOCl	19.1 ± 1.8^A,a^	18.1 ± 2.0^A,b^	17.4 ± 2.1^A,b^	17.4 ± 1.6^A,b^	17.4 ± 1.5^A,b^	17.4 ± 1.9^A,b^	17.5 ± 1.5^A,b^	17.9 ± 2.1^A,b^
G4 - 5% NaOCl	19.2 ± 0.9^A,a^	17.7 ± 1.0^A,b^	17.5 ± 1.1^A,b^	17.2 ± 1.0^A,b^	17.1 ± 0.8^A,b^	17.2 ± 1.3^A,b^	17.9 ± 1.0^A,b^	17.8 ± 1.4^A,b^

Different lowercase letters in rows indicate statistically significant intragroup differences (Two-way Anova and Tukey *post-hoc* P<0.05); Different capital letters in columns indicate statistically significant intergroup differences in the same time period (One-way Anova and Tukey *post-hoc* P<0.05)

## DISCUSSION

In the present study, the tissue dissolution capability and the changes in the dentin chemical composition by different concentrations of NaOCl solutions were assessed. The results demonstrated that NaOCl can dissolve the organic matter and deproteinate the collagen of dentin in high quantities; and otherwise, it can cause a small reduction in the carbonate component of the inorganic phase of the dentin.

The null hypothesis tested has to be rejected, since there were differences between the concentrations of NaOCl solutions in the ability of tissue dissolution and in the effects on dentin composition over time.

A concentration and time-dependent organic tissue dissolution capacity was observed for the NaOCl solutions (Table 1), as previously found in other studies^1^ ^,^ ^7^ ^,^ ^12^ ^,^ ^14^ ^,^ ^29^. NaOCl exerts a nonspecific, non-coagulating digestive effect on vital and necrotic tissues^14^ ^,^ ^26^ by direct contact between free available chlorine molecules and organic matter^20^. The pH of the solution influences the biological effects of NaOCl by determining the equilibrium of the freely available chlorine^4^, i.e., the sum of concentrations of hypochlorous acid and hypochlorite anion (HOCl/OCl^-^)^4^ ^,^ ^5^. Acid solutions have a powerful bactericidal effect because of the prevalence of HOCl. The OCl^-^ has a powerful oxidative effect that promote higher tissue dissolution and is more abundant in alkaline solutions^4^. Previous studies did not find differences in the tissue-dissolving properties of NaOCl at the same concentrations and different alkaline pHs of 9 and 12^7^ ^,^ ^33^. Although there were differences between the pHs of the solutions tested (Table 1) they were small and may not influence the tissue dissolution capability of the irrigants.

Tissues from different sources were used in studies about the tissue dissolving ability of irrigation solutions^1^ ^,^ ^7^ ^,^ ^8^ ^,^ ^12^ ^,^ ^14^ ^,^ ^29^. Bovine muscle was chosen because of the availability and easier standardization of the specimens^29^ ^,^ ^30^. To prevent the confounding factors in the dissolution analysis, the specimens were prepared with similar mass and surface areas. The same temperature and volume of the solutions were used for all groups, and to simulate the solution flow in the root canal during the root canal preparation, the solutions were agitated in an ultrasonic tub.

The bovine incisor dentin has a similar structure and number of tubuli of human molar dentin^28^ and permits the achievement of a more standardized substrate for analyses. There are no differences between the mineral matrices of human and bovine dentin, and from the bovine collagen and demineralized human dentin, only differences in intensities of absorption bands are observed^3^ ^,^ ^6^. The samples were prepared as slices to maintain the natural structure and to prevent changes in tissue composition, because the grinding processes can cause water loss to the ambient, shift the wavenumber, and alter the intensity of the absorption bands^3^.

In the dentin vibrational spectrum, it is possible to observe bands related to water, to hydroxyapatite, that originate from the carbonate and phosphate groups and to the organic matrix from the groups present in the collagen such as amides I, II, and III^3^. The treatment of dentin showed that the NaOCl leads to concentration-dependent collagen depletion (Table 2). Although there are no statistical differences between the G2, G3, and G4 groups, the removal of the organic phase from the superficial subsurface of mineralized dentin was considerably more severe for the 5% NaOCl, with significant differences for the saline solution from the 5 min of immersion. This lower amide III/phosphate ratio was also observed for higher concentrations of NaOCl in other studies^2^ ^,^ ^15^ ^,^ ^35^. The NaOCl acts on the dentin creating deproteination channels that leads to a non-uniform effect^10^, leaving unbound hydroxyapatite and an apatite-rich and collagen sparse dentin subsurface^10^ ^,^ ^11^. The destruction of the dentin collagen matrix results in a less tough and more brittle substrate^10^ ^,^ ^18^ that might facilitate the fatigue crack propagation during cyclic stresses^16^ ^,^ ^34^ and increase the susceptibility of crown or root fracture^34^. The destructive effect of NaOCl on the dentin is irreversible and if the chelating agent is subsequently employed, it removes the collagen-depleted apatite phase and exposes the underlying destruction caused by NaOCl, which is morphologically perceived as canal wall erosion^35^.

In the present study, a time-dependent effect in the reduction of the amide III/phosphate ratio was identified in all NaOCl concentrations (Table 2). The results indicate that there was a slow and continuous degradation of collagen from the dentin surface and they are in accordance with previous studies that also observed that the removal of the organic phase from the dentin is time-dependent^34^ ^,^ ^35^. Other studies reported an initial reduction in the collagen with a plateau in dentin deproteination reached over time for the same NaOCl concentration^2^ ^,^ ^10^ ^,^ ^15^ ^,^ ^21^. The plateau was not observed in this research; however, there was a reduction in the rate of deproteination over time. This reduction may be related to the fact that the collagen present on the dentin surfaces is quickly hydrolyzed and removed, and after the process it reverts to the deeper and unexposed collagen that is encapsulated by hydroxyapatite, being less vulnerable to the destructive effects of NaOCl and showing little changes over time^10^ ^,^ ^15^.

Carbonate groups may occupy phosphate and hydroxyl ions sites in bone and teeth apatite. These substitutions affect the crystallinity of the apatites and can accelerate the dissolution process of the tooth structure^23^ ^,^ ^31^. In the present study, a significant reduction in the carbonate/phosphate ratio occurred in all NaOCl concentrations tested after 0,5 min of immersion, but a plateau was observed after this immersion period (Table 3). Since the solutions were renewed to ensure their effectiveness, this plateau probably occurs after the removal of carbonate from the surface, because the accessibility to the groups that are in subsurfaces layers of dentin makes them less susceptible to the action of the NaOCl solutions. These results confirmed that carbonate groups are more soluble than phosphate groups^23^ ^,^ ^31^ and are in accordance with a previous study that also observed that the NaOCl treatment removes some carbonate ions from the inorganic dentin structure, while at the same time it deproteinates the organic matter^27^. This is consistent with the very low solubility expected for apatite mineral in alkaline solution^11^ and may be potentiated by the ultrasonic agitation.

The design of the present study does not directly reflect the clinical conditions, but allows quantitative evaluations regarding the different concentrations and time exposure of NaOCl solutions. This study confirmed the advantage of using a longer contact time and higher concentrations of NaOCl to promote tissue dissolution. However, it increases the alterations in dentin composition and the risks of periapical tissue damage from inadvertent extrusion. Based on these results, the use of NaOCl at lower concentrations, such as 1 and 2.5%, demonstrates to be effective in promoting a suitable dissolution of organic tissue present in the root canal system and preventing a pronounced damage to the dentin structure.

## CONCLUSIONS

The findings of this study indicated that the increase in the exposure time and in the concentration of NaOCl solution lead to an increase in the tissue dissolution and dentin collagen deproteination. Moreover, some carbonate ions are removed from the dentin inorganic phase by the NaOCl.
